# Supplementary material for: The impact of reduced skeletal muscle mass at stroke onset on 3-month functional outcomes in acute ischemic stroke patients
Source: PLoS One. 2025 Jan 15;20(1):e0313368. doi: 10.1371/journal.pone.0313368 (PMC11734988; doi:10.1371/journal.pone.0313368)
Supplement: S1 Fig — (DOCX) [file pone.0313368.s001.docx]

S1 Fig. Flow sheet showing the study design and patients exclusion criteria.


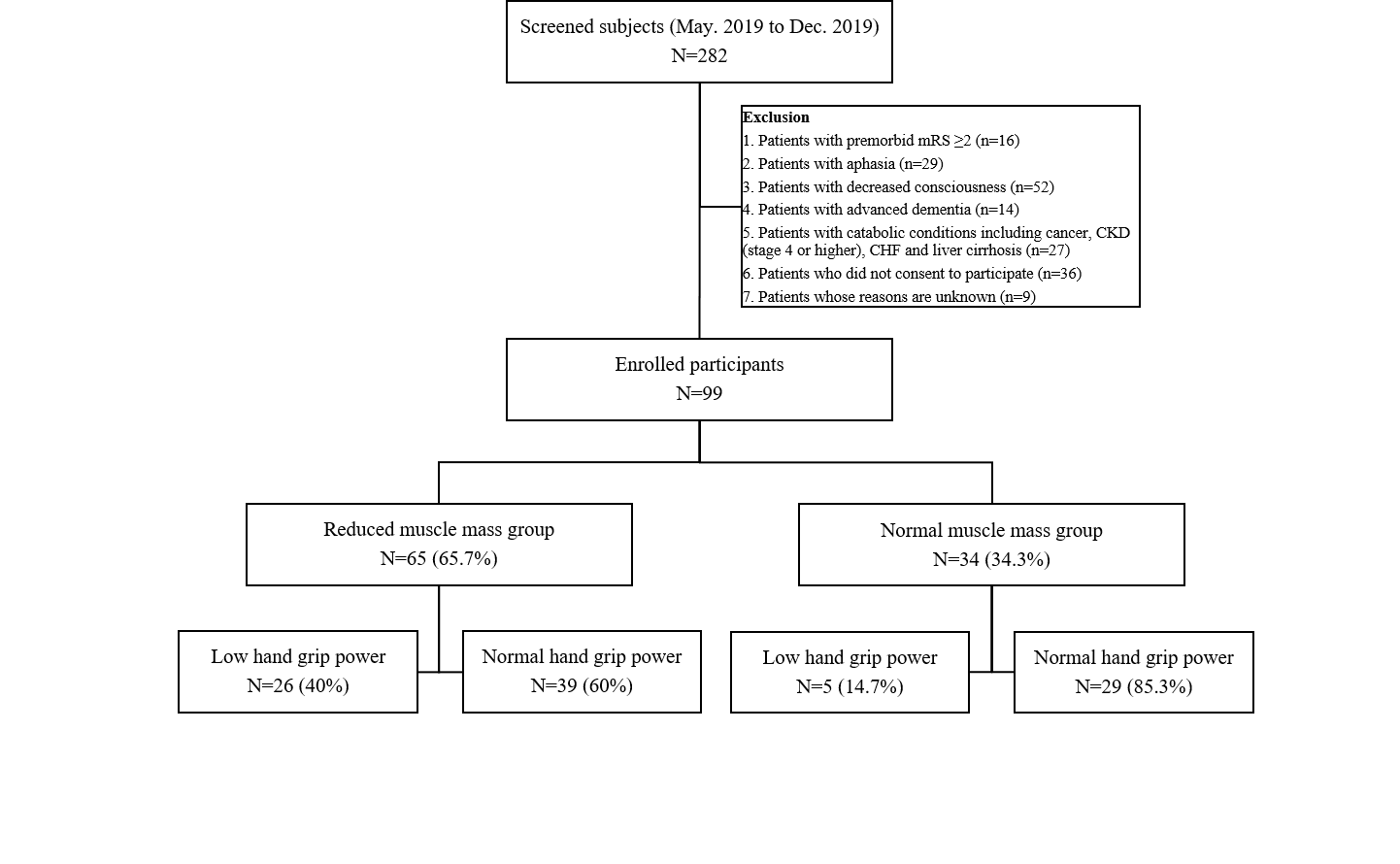


mRS : modified Rankin Scale, CKD : chronic kidney disease, CHF : congestive heart failure
