# Supplementary material for: The impact of reduced skeletal muscle mass at stroke onset on 3-month functional outcomes in acute ischemic stroke patients
Source: PLoS One. 2025 Jan 15;20(1):e0313368. doi: 10.1371/journal.pone.0313368 (PMC11734988; doi:10.1371/journal.pone.0313368)
Supplement: S1 Table — (DOCX) [file pone.0313368.s002.docx]

S1 table. Comparison of Laboratory Findings and Clinical Characteristics Between Reduced and Normal Muscle Mass Groups in Stroke Patients

|  | RMM (n=65) | NMM (n=34) | *p*-value |
| --- | --- | --- | --- |
| Age, mean (SD), years | 66.4 (11.7) | 59.97 (10.6) | 0.009 |
| BMI, mean (SD), kg/m^2^ | 23.4 (2.7) | 26.7 (2.8) | <0.001 |
| Male gender, n (%) | 52 (80) | 23 (62.7) | 0.173 |
| NIHSS on admission, median [IQR] | 3 [1-6] | 2 [0-5] | 0.015 |
| Onset-to-Door time, median [IQR], hours | 7.34 [2.48] | 5.27 [2.08] | 0.145 |
| Recanalization therapy, n (%) |  |  | 0.976 |
| None | 52 (80) | 27 (79.41) |  |
| IV tPA only | 5 (7.69) | 3 (8.82) |  |
| IAT only | 5 (7.69) | 2 (5.88) |  |
| Combined | 3 (4.62) | 2 (5.88) |  |
| SBP, initial, mean (SD), mmH_2_O | 165.1 (30.9) | 177.03 (30.4) | 0.069 |
| DBP, initial, mean (SD), mmH2O | 87.6 (15.6) | 95.1 (15.4) | 0.025 |
| Risk factors, n (%) |  |  |  |
| HTN | 41 (63.1) | 21 (61.8) | 0.898 |
| DM | 22 (33.9) | 9 (26.5) | 0.452 |
| Dyslipidemia | 34 (52.3) | 20 (58.8) | 0.536 |
| Coronary heart disease | 7 (10.8) | 2 (5.9) | 0.714 |
| Atrial fibrillation | 13 (20) | 4 (11.8) | 0.404 |
| Previous TIA or Stroke | 14 (21.5) | 2 (5.9) | 0.049 |
| Smoking within 5 years | 28 (43.1) | 14 (41.2) | 0.856 |
| TOAST classifications, n (%) |  |  | 0.346 |
| LAA | 24 (36.9) | 9 (27.3) |  |
| SVO | 19 (29.2) | 13 (39.4) |  |
| CE | 12 (18.5) | 3 (9.1) |  |
| Others | 10 (15.4) | 8 (24.2) |  |
| Laboratory findings, mean (SD) |  |  |  |
| WBC counts, ×10^3^/μL | 8.02 (2.9) | 8.32 (2.3) | 0.61 |
| Hemoglobin, g/dL | 14.1 (1.9) | 13.9 (1.9) | 0.807 |
| Hematocrit, % | 40.58 (5.1) | 40.41 (4.8) | 0.869 |
| Platelet counts, ×10^3^/μL | 221.3 (88.7) | 245.5 (68.5) | 0.167 |
| Total cholesterol, mg/dL | 188.8 (50.1) | 199.1 (41.1) | 0.305 |
| Triglyceride, mg/dL | 136.7 (74.2) | 144.1 (81.4) | 0.652 |
| HDL, mg/dL | 45.7 (11.1) | 45.1 (10.2) | 0.822 |
| LDL, mg/dL | 106.4 (32.1) | 115.4 (29.02) | 0.175 |
| Laboratory findings, median [IQR] |  |  |  |
| Fasting glucose, mg/dL | 125 [108-152] | 129 [107-160] | 0.935 |
| HbA1c, % | 6 [5.5-6.4] | 6.1 [5.7-6.3] | 0.845 |
| BUN, mg/dL | 16.1 [12.5-19] | 14.9 [12.4-21.3] | 0.712 |
| Creatinine, mg/dL | 0.79 [0.68-0.99] | 0.81 [0.68-1.01] | 0.822 |
| Cockcrofr-Gault eGFR, mL/min | 90 [80-97] | 91.5 [75-105] | 0.519 |
| AST, U/L | 25 [20-30] | 24.5 [20-29] | 0.774 |
| ALT, U/L | 19 [15-26] | 25.5 [17-38] | 0.044 |
| ESR, mm/h | 9 [5-17.5] | 9 [3-19] | 0.699 |
| hs CRP, mg/L | 1.2 [0.5-2.7] | 1.3 [0.55-2.55] | 0.868 |
| INR | 0.99 [0.94-1.04] | 0.96 [0.92-0.99] | 0.032 |
| aPTT, seconds | 33.9 [31.8-36] | 32.1 [30.2-34.1] | 0.043 |
| Fibrinogen, mg/dL | 324 [292-365] | 322 [295-368] | 0.851 |
| NT-proBNT, pg/mL | 116 [60-392] | 81 [30.5-215] | 0.218 |
| Outcomes, n (%) |  |  |  |
| mRS 0-1 at discharge | 35 (53.9) | 31 (91.2) | <0.001 |
| mRS 0-1 at 90 days | 37 (56.9) | 32 (94.1) | <0.001 |
| MACE¶ | 2 (3.08) | 2 (5.88) | 0.605 |

RMM: Reduced Muscle Mass, NMM: Normal Muscle Mass, SD: Standard Deviation, BMI: Body Mass Index, NIHSS: National Institutes of Health Stroke Scale, IQR: Interquartile Range, IV tPA: intravenous tissue plasminogen activator, IAT: intra-arterial therapy, SBP: Systolic Blood Pressure, DBP: Diastolic Blood Pressure, HTN: Hypertension, DM: Diabetes Mellitus, TIA: Transient Ischemic Attack, TOAST: Trial of Org 10172 in Acute Stroke Treatment, LAA: Large Artery Atherosclerosis, SVO: Small Vessel Occlusion, CE: Cardioembolism, WBC: White Blood Cells, HDL: High-Density Lipoprotein, LDL: Low-Density Lipoprotein, HbA1c: Hemoglobin A1c, BUN: Blood Urea Nitrogen, eGFR: Estimated Glomerular Filtration Rate, AST: Aspartate Aminotransferase, ALT: Alanine Aminotransferase, ESR: Erythrocyte Sedimentation Rate, hsCRP: High-Sensitivity C-Reactive Protein, INR: International Normalized Ratio, aPTT: Activated Partial Thromboplastin Time, NT-proBNP: N-terminal pro b-type Natriuretic Peptide, RHI: Reactive Hyperemia Index, LnRHI: Natural Logarithm of the Reactive Hyperemia Index, mRS: Modified Rankin Scale, MACE: Major adverse cardiovascular events

¶ In the RMM group, one patient experienced a recurrent ischemic stroke, and another had a cardiovascular event. In the NMM group, both cases were recurrent ischemic strokes.
